# Detecting High-Risk Factors and Early Diagnosis of Diabetes Using Machine Learning Methods

**DOI:** 10.1155/2022/2557795

**Published:** 2022-09-29

**Authors:** Zahid Ullah, Farrukh Saleem, Mona Jamjoom, Bahjat Fakieh, Faris Kateb, Abdullah Marish Ali, Babar Shah

**Affiliations:** ^1^Department of Information Systems, Faculty of Computing and Information Technology, King Abdulaziz University, Jeddah, Saudi Arabia; ^2^Department of Computer Sciences, College of Computer and Information Sciences, Princess Nourah Bint Abdulrahman University, Riyadh, Saudi Arabia; ^3^Department of Information Technology, Faculty of Computing and Information Technology, King Abdulaziz University, Jeddah, Saudi Arabia; ^4^Department of Computer Science, Faculty of Computing and Information Technology, King Abdulaziz University, Jeddah, Saudi Arabia; ^5^College of Technological Innovation, Zayed University, Abu Dhabi, UAE

## Abstract

Diabetes is a chronic disease that can cause several forms of chronic damage to the human body, including heart problems, kidney failure, depression, eye damage, and nerve damage. There are several risk factors involved in causing this disease, with some of the most common being obesity, age, insulin resistance, and hypertension. Therefore, early detection of these risk factors is vital in helping patients reverse diabetes from the early stage to live healthy lives. Machine learning (ML) is a useful tool that can easily detect diabetes from several risk factors and, based on the findings, provide a decision-based model that can help in diagnosing the disease. This study aims to detect the risk factors of diabetes using ML methods and to provide a decision support system for medical practitioners that can help them in diagnosing diabetes. Moreover, besides various other preprocessing steps, this study has used the synthetic minority over-sampling technique integrated with the edited nearest neighbor (SMOTE-ENN) method for balancing the BRFSS dataset. The SMOTE-ENN is a more powerful method than the individual SMOTE method. Several ML methods were applied to the processed BRFSS dataset and built prediction models for detecting the risk factors that can help in diagnosing diabetes patients in the early stage. The prediction models were evaluated using various measures that show the high performance of the models. The experimental results show the reliability of the proposed models, demonstrating that k-nearest neighbor (KNN) outperformed other methods with an accuracy of 98.38%, sensitivity, specificity, and ROC/AUC score of 98%. Moreover, compared with the existing state-of-the-art methods, the results confirm the efficacy of the proposed models in terms of accuracy and other evaluation measures. The use of SMOTE-ENN is more beneficial for balancing the dataset to build more accurate prediction models. This was the main reason it was possible to achieve models more accurate than the existing ones.

## 1. Introduction

Diabetes mellitus is a metabolic disease caused by the presence of an excessive amount of glucose in the blood due to the inadequate secretion of insulin or insulin resistance [1]. The pancreas is the main source for producing insulin, a crucial hormone that is responsible for transferring the converted glucose through the bloodstream to different body parts [2]. Furthermore, the inappropriate secretion of insulin causes the glucose to persist in the blood, which ultimately causes a surge in the sugar level in the blood [2]. This disease causes a huge economic burden and has attracted deep public concern globally [3]. According to [4], diabetes has hugely burdened the US economy, with a total estimated cost of 327 billion in 2017, including the direct medical cost of 237 billion and 90 billion in reduced productivity. It is evident from several estimations and forecasts that diabetes is related to augmented mortality and has increasing prevalence [5]. As per the report of [6] discussed in [3], the worldwide prevalence of diabetes was around 9.3% in 2019 among adults, accounting for a total of around 463 million adults with diabetes; the report further predicted that this number may increase to 700 million in 2045. According to a report [7], around 422 million people have diabetes globally, of whom the majority live in low and middle income countries, and around 1.5 million mortality cases are due to diabetes every year.

Diabetes has three different types: type 1, type 2, and gestational [2, 4]. In most cases, patients recover from gestational diabetes after delivery, while prediabetes can be controlled through proper diet and exercise [2]. Type 1 diabetes is mostly detected in people under 30 years of age [8]. However, type 2 diabetes develops at a later age [4] due to obesity and insulin resistance of cells [2], high blood pressure, dyslipidemia, arteriosclerosis, and other related diseases [8]. In addition to these risk factors, recent experiments show that some environmental endocrine disturbances might cause the occurrence of diabetes [3]. Among the types of diabetes, type 2 is predictable and preventable because it occurs at a later age due to lifestyle and other risk factors [4].

Diabetes is a common disease that affects people worldwide and increases the risk of life-threatening long-term complications such as heart disease and kidney disease, among others [9]. However, if diabetes is detected at an early stage, patients can live longer and healthier. Approaches of artificial intelligence (AI) and machine learning (ML) have changed and affected every sector. Generally, the medical sector is one of the vital sectors where healthcare makes great use of such technology in terms of detecting and diagnosing some critical diseases [10, 11]. One of them is the use of ML to identify the risk factors of diabetes at the early stage and diagnose the disease before complications occur. While ML methods have increased the accuracy of medical diagnosis while reducing medical costs [12] of diagnosing and without surgical intervention. In the literature, several attempts have been made to detect and diagnose diabetes.

This study aims to develop prediction models for detecting the risk factors that cause diabetes and to provide decision-based models for diagnosing this disease at an early stage. For this purpose, several ML techniques are used to provide an accurate model that can help medical practitioners in diagnosing this disease. The experimental results show the higher performance of the proposed models in terms of accuracy and other evaluation measures. The better performance of the proposed models provides support for using these models as a decision support system to detect the risk factors of diabetes and help medical doctors in diagnosing diabetes mellitus at an early stage.

The rest of this study is organized as related work has been described in the next section, followed by a detailed methodology.  Section 4 describes the experimental setup; Section 5 describes the results and discussion.  Section 6 concludes this study.

## 2. Related Work

In this section, domain-specific studies are analyzed to understand the trends and techniques used in the existing studies for detecting the high-risk factors of diabetes using ML methods. For this purpose, several databases were explored with various keywords for searching related studies. The databases searched included Google Scholar, Science Direct, IEEE Xplore, MDPI, and several others. In the existing studies, most of the researchers have used the Pima India diabetes dataset (PIDD) for detecting, diagnosing, early diagnosing, building smart applications, and other functions for diabetes patients. For example, in [8], two datasets (i.e., a private dataset and the PIDD) were used. The authors used principal component analysis (PCA) and minimum redundancy maximum relevance (mRMR) for the dimensionality reduction. Several ML algorithms were used for detecting diabetes. The results reported that RF outperformed other methods with an accuracy of 80.84% for the private dataset, while the PIDD yields an accuracy of 77.21%. Similarly, [13] attempted to detect diabetes patients using ML methods. They used the PIDD and used the PCA methods for dimensionality reduction. A bootstrapping method was used to compare the performance of the trained models. The reported results show better performance of SVM and AB classifiers after the bootstrap operation that both achieved an accuracy of 94.44%.

Reference [4] attempted to build risk prediction models for type 2 diabetes. They used the BRFSS-2014 dataset and trained several ML models. In the dataset, the class imbalance issue was handled using the SMOTE method in order to avoid bias. The experimental results showed that the overall performance of the neural network (NN) showed a higher accuracy rate of 82.41% than all other measures.

In [14], the authors proposed a comparative study of ML methods for the efficient diagnosis of five major diseases, including diabetes. The authors used the BRFSS dataset and trained logistic regression and RF models based on it. The theme of the study is to predict the percentage of chronic diseases based on the inputs via a chatbot in which suggestions are provided using modeled and interactive data visualization to lower the risk. They have attempted several experiments with different parameters and concluded that RF with 100 trees and a maximum depth of 10 achieved better results than LR, detecting diabetes with an accuracy of 86.80%.

In [15], the authors used 24 different classification algorithms for detecting diabetes in the early stage. The experiment was performed using MATLAB. The model performance was evaluated using cross-validation. The authors reported that the LR was the best fitted model of all 24 ML methods used in the study, as LR reached an accuracy rate of 77.9%.

A study conducted by [16] used the PIDD and trained 7 different ML models. In this approach, a feature selection was used in which two of the features were dropped. The highest accuracy of LR and SVM reached around 77%-78% in both split and k-fold validations. The same dataset was also used for training the NN model with different hidden layers, learning rates, and iterations. The authors concluded that NN with 2 hidden layers outperformed other methods with an accuracy rate of 86.6%.

An attempt was made by [9] to detect diabetes using ML methods. In this study, the authors used two datasets (i.e., the PIDD and another dataset) and applied several ML algorithms. Various preprocessing steps, such as label encoding and normalization, were utilized for improving the accuracy rate of the prediction models. The author reported that SVM outperformed the rest of the methods with an accuracy rate of 80.26% on the PIDD, while DT and RF outperformed the other datasets with an accuracy rate of 96.81%. Based on the prediction model, the author developed a smart web application.

The authors of [17] used the PIDD for predicting diabetes using ML methods. A total of five ML algorithms were applied to the processed data, with two additional extracted features. The models were trained using the split method, with 70% of the data used for training and the remaining 30% used for testing. The model's performance was measured using evaluation measures. The reported results reached the highest accuracy rate for the RF model at 88.31%.

The risk factors for diabetes are outlined in [2] using ML techniques. The data collection was carried out using a survey distributed randomly to Indian participants, and 251 responses were received. Three ML algorithms were used: LR, SVM, and RF. The reported results show that LR outperformed the other two methods and achieved an accuracy rate of 96.02%. Likewise, a study conducted by [18] applied various machine learning algorithms to a dataset consisting of 520 observations containing data about both new and diabetic patients. The experimental results exhibited higher accuracy achieved by the bagged method, at 97.7%.

A novel approach of hybrid firefly bat optimized fuzzy artificial neural network (FFBAT-ANN) was proposed by [19] for diagnosing diabetes. In this approach, the fuzzy rules were produced using the LPP method by identifying the features related to diabetes, and the classification was performed using the FFBAT-ANN method. The reported results show the high performance of the proposed method in that FFBAT-ANN achieved a higher accuracy rate of 74.4%.  Table 1 summarizes the related work.

## 3. Methodology

This section will discuss the step-by-step methodology used for conducting this study. Data analysis was performed using Python. The rest of the steps will be discussed in the following subsections.

### 3.1. Data Collection

**Figure 1:**
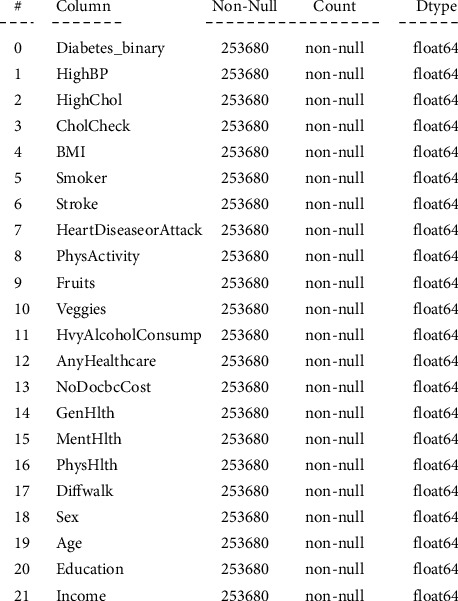
Dataset description.

The data collection was carried out from the publicly available data source Kaggle [20], which was collected from the behavioral risk factor surveillance system (BRFSS) [21]. The collected data is a cleaned version of the BRFSS, which consists of a total of 253,680 records reflecting the actual responses to the survey conducted by the CDC's BRFSS2015. The dataset comprised a total of 22 features, including the class feature. The class variable (Diabetes_binary) is a binary variable indicating whether the patient has diabetes. More specifically, “0” indicates no diabetes, and “1” indicates prediabetes or diabetes. Moreover, this study used the whole feature set for training the proposed models.  Figure 1 shows the features of the dataset.

### 3.2. Data Preprocessing

One of the challenging steps in building prediction models, and especially healthcare decision support systems, is to prepare the data in a manner conducive to the achievement of reliable results. The raw data collected from real-world scenarios is often incomplete, imbalanced, and not clean [22, 23]. Therefore, before training the model with real-world data, various preprocessing steps must be used to enhance the quality of the data [24]. ML provides several methods for cleaning the data. For example, the missing values can be handled with imputers, etc. In this study, several steps were utilized for handling the inconsistencies in the dataset.

**Figure 2:**
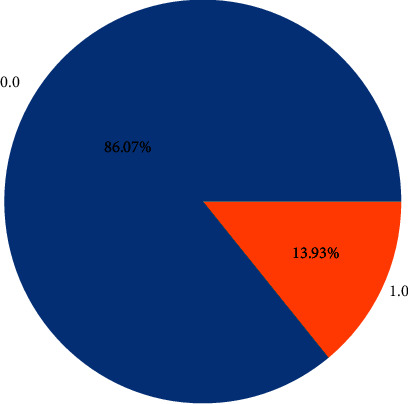
Imbalanced dataset.

Although the data has no missing values, the dataset was extremely imbalanced, as shown in Figure 2. In an imbalanced data scenario, the data of a certain type are fewer in number than the other types of data in a dataset [25]. Most of the time, the minority class type is of interest for investigation. In Figure 2, the class labeled “0.0” represents 86.07% of the data, while the class labeled “1” accounts for only 13.93%. To balance the class types in a dataset, researchers use various methods, such as the SMOTE [26], random oversampling, and other subtypes. In the SMOTE method, the minority class is oversampled in which the minority class samples are considered and generate synthetic samples in the feature area based on the selected *k* number in the KNN [27].

In this study, the imbalanced dataset problem was handled using SMOTE-ENN. SMOTE-ENN [28] is a powerful method that merges the advantages of both SMOTE and ENN, with SMOTE oversampling the minority class and ENN undersampling the majority class samples [25]. Moreover, ENN drops any samples whose class types are different from the class of at least two of its three nearest neighbors; hence, any sample that is inaccurately classified by its three nearest neighbors is dropped from the training dataset [29]. The application of SMOTE-ENN for handling the imbalanced dataset problem achieved better performance than the single SMOTE method. Similarly, the dataset was normalized using feature scaling, in which the data were transformed between 0 and 1. Feature scaling is a useful method for enhancing model accuracy.

### 3.3. Prediction Models

In this study, various ML models were applied to the BRFSS dataset. For the building of each model, hyperparameter tuning was performed to choose the best fitted set of parameters that are optimal for achieving the best performance of the model. The models achieved high performance in terms of accuracy, and other evaluation measures were finalized for predicting the high-risk factors of diabetes. The following section discusses the finalized prediction model for this study.

#### 3.3.1. KNN

KNN is an ML method that classifies the data based on the nearest proximity of training data in a feature set [30]. In this method, the classifier attempts to find the *k* number of closely similar samples from the training set for predicting the class label of a new sample. Furthermore, the *k* number is set to an odd number, which ensures that the majority of a class is recognized clearly [31]. In this method, the *k* number is set to 3 to achieve higher accuracy and other evaluation measures.

#### 3.3.2. RF

RF is an ensemble machine learning technique that utilizes several DT to create a forest. In this method, each DT in the forest is trained using randomly selected training data and a subset of features [31]. Moreover, the main parameter for this method is the number of trees [32]. The majority of trees selected by the RF are the ultimate selection of the classification [33]. In this study, the number of trees was set at 50 for building the RF model. The model evaluation shows the higher performance of the RF model with the best-fitted parameters.

#### 3.3.3. XGBoost

XGBoost (XGB) is a recently developed ML algorithm proposed by Chen and Guestrin [34] in 2016. This is an enhanced algorithm based on gradient boosting DT that can significantly build boosted trees and execute them in parallel [35]. In the iteration process, gradient boosting seeks to enhance the robustness by dropping the loss function of the algorithm as well as the gradient direction [25]. XGB trains multiple classifiers slowly and sequentially. Like RF, the boosting algorithm is using DT, but it depends on individuals how to utilize them [36]. In this study, the number of trees was set to 100 based on the suggested hyperparameter tuning test for building the XGB model.

#### 3.3.4. Bagging

Bagging is an ensemble learning method combining several classifiers using training data, in which different training data are presented for learning in each instance. Moreover, the new training set is generated by randomly selected examples with replacements from the original training set. A class achieving the majority of votes wins [37]. Moreover, in this method, several trees using a bootstrap sampling of the training set are created and integrated into their individual predictions to achieve the final classification. In this study, the number of trees per hyperparameter tuning is set to 100 with the bootstrap method. The model shows higher performance in terms of accuracy and other evaluation measures.

#### 3.3.5. AB

AB is an ensemble ML method that aims to integrate several weak classifiers and transform them into strong ones [38]. In this method, DT is used as a default base estimator for training the model. The base estimator in AB is a weak learner in which every tree is trained to reduce the weakness by learning from the trees being trained that are boosted using weights. Moreover, this is a loop-based method in which weights are assigned to train the data in every iteration of the loop. The iteration process continues until the accurate classification of the data is confirmed [37]. Per the hyperparameter tuning, the number of trees was set to 100 for building the AB model.

### 3.4. Model Evaluation

Model evaluation is the practice of measuring the prediction results of the model built and then comparing those results against the real data, which is generally known as test data [39]. For model evaluation, there are several methods available, but this study utilized the percentage split method. In this method, the processed dataset was split into two sets; 70% of the whole dataset was used for training the aboveproposed models, and the remaining 30% was used for testing the efficacy of the proposed models. The model evaluation shows the higher performance of the proposed model.

## 4. Experimental Results

### 4.1. Experimental Setup

The prediction models discussed in the above sections were applied to the BRFSS dataset for detecting the risk factors associated with diabetes, which can be useful for diagnosing diabetes in patients at an early age. As noted above, the dataset was initially split into two subsets; the training set comprised 70% of the total dataset, while the remaining 30% was used as the testing set. During the experiment, several attempts were made to finalize the best classifiers to accurately detect the risk factors. Therefore, a hyperparameter test was utilized to set the most suitable parameters of each classifier to maximize the likelihood of predictions in terms of selecting an accurate model that can help medical practitioners in decision-making about diabetes patients. After running several experiments with best fitted parameters on the processed data, and the best classifiers according to accuracy and other measures were used to report the results.

**Figure 3:**
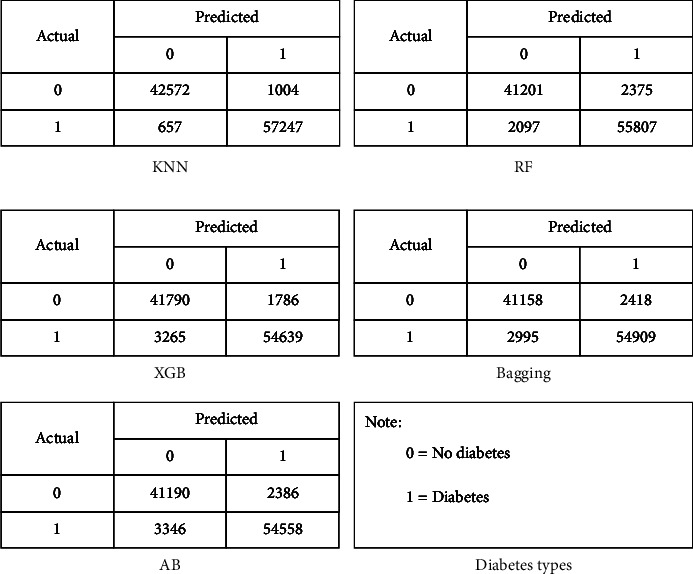
Confusion matrix.

In the experimental phase, for building each model, a confusion matrix is computed, which provides four important values: true-positive (tp), true-negative (tn), false-positive (fp), and false-negative (fn), as shown in Figure 3. The model evaluation was performed on the basis of these four values using the following measures:

(i) Accuracy is the ratio of correctly identified diabetes patients to the whole number that is predicted [40]. Equation (1) shows the mathematical representation of accuracy.
  (1) $$\begin{array}{c} Accuracy=\frac{tp+tn}{tp+tn+fp+fn}\text{.} \end{array}$$
(ii) Precision, a measure calculated using equation (2), is the ratio of correctly identified patients with diabetes to all patients with diabetes [41].
  (2) $$\begin{array}{c} Precision=\frac{tp}{tp+fp}\text{.} \end{array}$$
(iii) Recall or sensitivity, calculated using equation (3), is the ratio of correctly classified diabetes patients to the whole numbers in that particular class [41].
  (3) $$\begin{array}{c} RecallorSensitivity=\frac{tp}{tp+fn}\text{.} \end{array}$$
(iv) *F*-measure is the weighted average of precision and recall [40] and is mathematically calculated using .
  (4) $$\begin{array}{c} F-measure=\frac{\left(2*Precision*Recall\right)}{Precision+Recall}\text{.} \end{array}$$
(v) Specificity is a performance measure of a model that is defined as the ratio of correctly classified patients without diabetes to all patients who do actually have diabetes [41]. Specificity is also known as true-negative rate (TNR).
(vi) ROC is a visualized curve that measures the performance of classifiers at various thresholds, while the AUC is a measurement of separability between the class labels. A higher AUC value shows a higher performance of the model in terms of accurately differentiating between patients with and without diabetes [40].

## 5. Results and Discussion

Comparing the experimental results of the proposed method to the existing state-of-the-art methods in the literature, our proposed method showed high performance in terms of accuracy, precision, sensitivity, specificity, f-measure, and ROC/AUC score.  Table 2 shows the comparison of the proposed method to prominent existing studies using the BRFSS dataset. Although the proposed prediction models showed higher performance compared to the existing, Table 2 reported the KNN results in the comparison table.

On the BRFSS dataset, our proposed method showed higher performance than the existing methods in that KNN achieved an average test accuracy of 98.363%; precision, sensitivity, and *f*-measures of 98%; and ROC/AUC score of 98.3%, which are the highest values so far. The reason the proposed methods were able to achieve high accuracy and other evaluation measures is the use of the SMOTE-ENN method, which is used for balancing the dataset in the preprocessing step. The SMOTE method alone was also tested on the BRFSS dataset, but the performance of the proposed models was not much different from that found in the existing studies. Therefore, the use of SMOTE-ENN is more powerful than the SMOTE method alone.

Similarly, our KNN method also outperformed those of other studies that used other prominent datasets, such as PIDD and other private datasets, as shown in Table 3. This shows the reliability of our proposed method for predicting the risk factors of diabetes.

**Figure 4:**
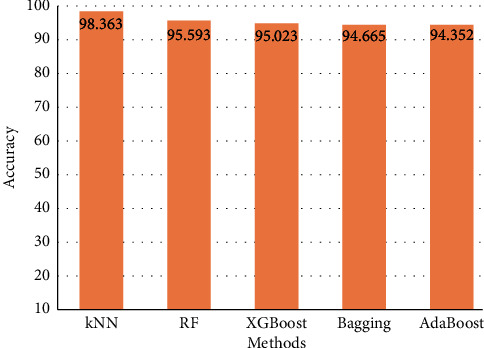
Accuracy of the proposed methods.

Moreover, the individual performance of each proposed method with a detailed discussion is shown in the following tables and figures.  Figure 4 shows the accuracy of the proposed methods in predicting the high-risk factors for detecting and diagnosing diabetes patients at an early stage.

Moreover, the proposed methods were also evaluated using precision, sensitivity, specificity, f-measure, and AUC scores. Precision, which is also referred to as positive predictive value (ppv), here refers to the fraction of accurately classified patients having diabetes over the total number of patients who actually have diabetes [41, 42]. The precision is also called the confidence of the prediction model. Sensitivity is the fraction of accurately classified patients with diabetes over the total number of patients in that class [40]. The F-measure is the harmonic mean of ppv and sensitivity [41].  Table 4 shows the model evaluation measures.

The values in Table 4 are the average measures for a model evaluation that surpasses the values in the comparison in Table 2, which shows the reliability of the proposed models in detecting diabetic patients to help medical practitioners in diagnosing the patients at an early stage.

**Figure 5:**
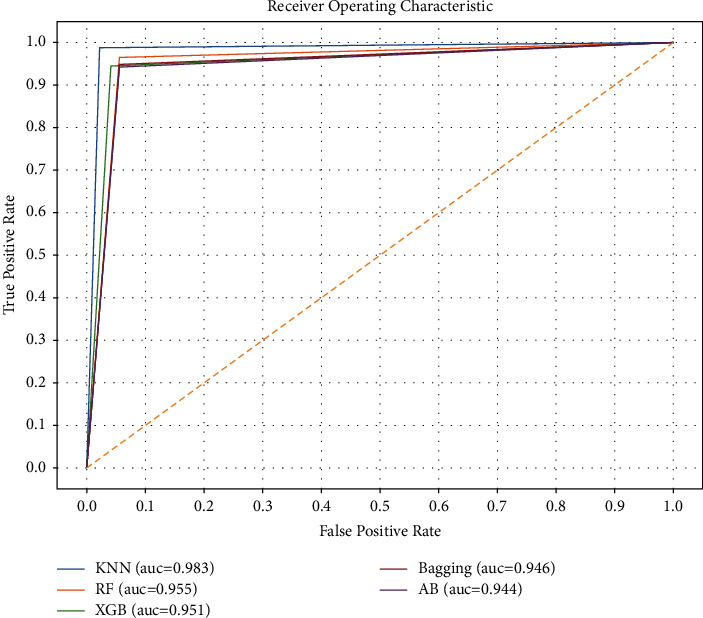
ROC curves of prediction models.

Similarly, the model was also evaluated using the ROC curves. ROC curves are highly beneficial for creating classifiers and visualizing their performance and are commonly utilized in healthcare decision-making [37], because they envisage the whole scenario of the trade-off between sensitivity and false-positive rate across a set of thresholds and are considered a powerful measure of a diagnostic test [43]. In the ROC, the AUC values decide the performance of a model. The higher the AUC score, the higher the performance of a prediction. An AUC value close to the left upper corner shows the high performance of the model. The AUC score shown in Table 4 is high, as it is very close to the left upper corner, and this is reflected in the ROC graph, as shown in Figure 5.

To summarize the above discussion, it is essential to prepare the data in a high-quality manner, especially for prediction purposes. Predictions are actually based on historical data from which the hidden patterns are extracted to form the basis for predicting the unseen cases. Therefore, the historical data should be of high quality, especially when the predictions are made in the healthcare field, where lives are at high risk. For these reasons, several preprocessing steps must be performed to remove outliers, handle the missing values, and balance the data in a manner that allows for the building of high-quality prediction models that can help medical practitioners in deciding about a particular disease.

The dataset used in this study was preprocessed in advance but was extremely imbalanced. The data imbalance issue was handled using SMOTE-ENN, which is a more powerful method than the SMOTE method alone. Thus, several ML algorithms were applied to the processed data. For the building of each model, hyperparameter tuning was performed to choose the best fitted model architecture for detecting the high-risk factors of diabetes. After running several experiments with optimal model architecture on the processed data, and the best classifiers according to accuracy and other measures were used to report the results. In this study, the finalized classifiers for detecting the high-risk factors of diabetes are KNN, RF, XGBoost, Bagging, and AdaBoost. The results achieved by these models were also compared to the existing state-of-the-art studies, and the efficacy of our proposed methods was found to be higher in terms of testing accuracy, precision, sensitivity, f-measure, and ROC/AUC score. This shows that the proposed models can be used as a decision-making process for detecting high-risk factors for diabetes and can also help medical practitioners in diagnosing diabetes patients in the early stages.

## 6. Conclusion and Future Work

This study was conducted to provide a system that can automatically detect the risk factors of diabetes as well as to provide an automatic decision-making system that can help medical practitioners in diagnosing diabetes patients based on risk factors. For that purpose, various preprocessing methods were used to prepare the data to increase the likelihood of prediction and increase the opportunity for developing reliable models. Moreover, hyperparameter tuning was performed for the building of each model to finalize the optimal parameter set that can achieve the maximum possible accuracies. Therefore, various experiments were performed on the processed BRFSS dataset in which the finalized methods discussed in the above sections achieved the best possible results in terms of accuracy, precision, sensitivity, specificity, f-measure, and ROC/AUC score. Among them, KNN outperformed the best-fitted model compared to others and even the state-of the art methods available in the literature. The reason behind the high performance of the proposed method was the use of the SMOTE-ENN method for handling the imbalanced dataset problem. The study has also attempted to use the SMOTE method alone, but the results were not much different from those of the existing studies. The use of SMOTE-ENN made it possible to achieve higher accuracies of the proposed models compared to the existing ones. This confirms the reliability of the proposed method for detecting the risk factors of diabetes as well as for providing accurate decision support systems for diagnosing diabetes early before it becomes chronic.

In the future, our model can be tested on other datasets collected from different clinics and research centers. The model efficiency can be enhanced using other advanced methods in the future.

## Figures and Tables

**Table 1:** Summary of related work.

S. No.	Ref.	Dataset	Preprocessing method(s)	Outperformed method(s)	Model accuracy (%)
1	[8]	Private	PCA, mRMR	RF	80.84
		PIDD			77.21
2	[13]	PIDD	PCA	SVM, AB, bootstrap	94.44
3	[4]	BRFSS-2014	SMOTE	NN	82.41
4	[14]	BRFSS	Different parameters used	RF	86.80
5	[15]	—	—	LR	77.9
6	[16]	PIDD	Feature selection	NN	86.6
7	[9]	PIDD	Label encoding, normalization	SVM	80.26
		Other		DT, RF	96.81
8	[17]	PIDD	Features extraction	RF	88.31
9	[2]	Private	—	LR	96.02
10	[18]	Private	—	Bagging	97.7

**Table 2:** Comparison of the proposed method with existing studies used BRFSS dataset.

Study	Dataset	Method	Accuracy (%)	Sensitivity	Specificity	AUC
[4]	BRFSS-2014	NN	82.4	0.378	0.902	0.795
[14]	BRFSS-2017	RF	86.8	—	—	—
Proposed method	BRFSS-2015	KNN	98.36	0.98	0.98	0.983

**Table 3:** Comparison of the proposed method with existing studies that used other datasets.

Study	Dataset	Method	Accuracy (%)	Precision	Sensitivity	Specificity	*F*-measure
[8]	Private	RF	80.84	—	0.85	0.767	—
	PIDD	RF	77.21		0.746	0.799	
[13]	PIDD	SVM, AB	94.44	0.971	0.910	—	—

[16]	PIDD	LR,SVM	78.85, 77.71	0.788, 0.774	0.789, 0.777	—	0.788,0.775
		NN	88.6	—	—	—	
[17]	PIDD	RF	88.31	0.88	0.86	—	0.87
[2]	Private	LR	96.02	0.887	0.857	—	0.871
Proposed method	BRFSS	KNN	**98.36**	**0.98**	**0.98**	**0.98**	**0.98**

**Table 4:** Model evaluation measures.

Classifier	Precision	Sensitivity	Specificity	*F*-measure	AUC
kNN	0.98	0.98	0.98	0.98	0.983
RF	0.96	0.95	0.95	0.95	0.955
XGBoost	0.95	0.95	0.96	0.95	0.951
Bagging	0.93	0.94	0.94	0.94	0.946
AdaBoost	0.94	0.94	0.95	0.94	0.944

## Data Availability

The data were taken from the publicly available data source Kaggle [20].
